# Engineering T7 RNA polymerase-cascaded systems controlled by nisin and theophylline for protein overexpression and targeted gene mutagenesis in *Lactococcus lactis*

**DOI:** 10.1016/j.synbio.2025.06.008

**Published:** 2025-06-22

**Authors:** Ying Huang, Kang Ma, Yan Li, Qingyan Li, Fuping Lu, Xueli Zhang, Zhe Sun

**Affiliations:** a College of Biotechnology, Tianjin University of Science and Technology, Tianjin, 300457, China; b Key Laboratory of Engineering Biology for Low-carbon Manufacturing, Tianjin Institute of Industrial Biotechnology, Chinese Academy of Sciences, Tianjin, 300308, China; c National Center of Technology Innovation for Synthetic Biology, Tianjin, 300308, China

**Keywords:** *Lactococcus lactis*, NICE system, Theophylline riboswitch, T7 RNA polymerase, Targeted mutagenesis

## Abstract

*Lactococcus lactis* serves as an important platform for heterologous protein production, with the nisin-controlled gene expression (NICE) system being widely employed for regulated protein overexpression. However, the NICE system relies on the native RNA polymerase, which limits transcriptional efficiency, and there remains a lack of tools enabling continuous target gene mutagenesis in *L. lactis*. In this study, we enhanced the NICE system by integrating the highly processive T7 RNA polymerase (T7RNAP) to boost protein expression. A theophylline-dependent riboswitch, RbxE, was incorporated into the nisin-induced promoter to mitigate the toxicity caused by basal T7RNAP expression in *Escherichia coli*. Directed mutagenesis of the riboswitch region between the stem-loop and the ribosome binding site optimized T7RNAP expression, leading to a 2.4-fold increase upon nisin and theophylline induction in *L. lactis.* The resulting NICE-T7 system achieved a 2.8-fold increase in GFP compared to the original NICE system. Furthermore, adenosine deaminase TadA8e was fused to T7RNAP to generate the MutaT7LL system, facilitating targeted A-to-G mutagenesis and successfully reactivated an erythromycin resistance gene with a mutation efficiency of 1.33 × 10^−6^. Overall, this study presents an upgraded NICE system that enhances protein production and enables continuous in vivo mutagenesis of target genes in *L. lactis*.

## 1 Introduction

*Lactococcus lactis* is a gram-positive lactic acid bacterium widely used in food fermentation and classified as generally recognized as safe (GRAS) [1,2]. Its efficient protein secretion capacity, limited extracellular protease activity, and GRAS status make *L. lactis* an attractive platform for heterologous protein production, particularly for vaccine antigens and pharmaceutical products [3,4]. Among available expression tools, the nisin-controlled gene expression system (NICE) is one of the most widely employed platforms in *L. lactis* for inducible gene overexpression and protein production [5]. The NICE system integrates the *nisK* and *nisR* genes, encoding a two-component regulatory system, into the chromosome of a nisin-negative *L. lactis* strain [6]. Upon nisin addition, the histidine kinase NisK undergoes autophosphorylation and transfers the phosphate group to the response regulator NisR. Activated NisR subsequently triggers transcription of target genes downstream of the *nisA* promoter [7]. The system enables over 1000-fold induction of protein expression using nisin, a food-grade inducer. However, this system relies on the native *L. lactis* RNA polymerase, which exhibits relatively low transcriptional efficiency, often limiting overall protein yield.

In contrast, T7 RNA polymerase (T7RNAP) from bacteriophage T7 exhibits a much higher transcription rate exceeding 200 nucleotides per second [8], enabling rapid mRNA accumulation and resulting in faster and higher protein production. Additionally, T7RNAP has high specificity for T7 promoter and does not transcribe host genes, minimizing unintended cellular stress [9,10]. These properties have made T7RNAP a widely adopted tool for high-level protein expression across various microorganisms, including *Escherichia coli* [11], *Pseudomonas putida* [12], *Bacillus subtilis* [13], and *Corynebacterium glutamicum* [14]. Notably, T7RNAP has also been utilized in *L. lactis* to express tetanus toxin fragment C [15]. Beyond protein overproduction, T7RNAP is employed as a transcriptional amplifier in synthetic circuits, where it converts weak input signals into strong transcriptional outputs. This amplification strategy enhances biosensor sensitivity [16,17] and has been applied in systems designed to increase transcriptional output by incorporating T7 promoters [18,19]. T7RNAP has also been integrated downstream of inducible promoters in *Ralstonia eutropha* to amplify protein expression [20]. These studies collectively highlight the potential of integrating T7RNAP to enhance the NICE system's protein production capacity in *L. lactis*.

Importantly, T7RNAP's orthogonality to host transcriptional machinery enables its application in in vivo continuous evolution platforms. By fusing base deaminases such as cytosine or adenosine deaminases to T7RNAP, engineered systems like MutaT7 facilitate targeted, continuous mutagenesis of genes placed downstream of T7 promoters. This strategy has been successfully demonstrated in *E. coli* [21], *C. glutamicum* [22], *Saccharomyces cerevisiae* [23], and other microorganisms. Compared to other continuous mutagenesis tools, such as EvolvR and OrthoRep, the MutaT7 system offers distinct advantages: it supports broader mutational windows, exceeding the ∼350 nucleotides achievable with EvolvR [24], and exhibits low host dependency, unlike the species-specific OrthoRep system [25]. While CRISPR-deaminase base editors have been developed in *L. lactis* by fusing cytosine or adenosine deaminases with dCas9, their editing is restricted to a 5-nt window size [26], making them unsuitable for continuous gene evolution applications. Furthermore, conventional in vitro mutagenesis methods, such as error-prone PCR, are challenging in *L. lactis* due to its low transformation efficiency [27]. As a result, efficient tools for in vivo continuous gene evolution in *L. lactis* remain limited and warrant development.

In this study, we engineered an upgraded NICE system designed to improve protein production and enable targeted gene mutagenesis in *L. lactis*. Specifically, T7RNAP was integrated into the NICE system, and a theophylline-dependent riboswitch was incorporated into the 5′ untranslated region to minimize leaky T7RNAP expression and mitigate toxicity in *E. coli*. The riboswitch was subsequently optimized through mutagenesis to fine-tune T7RNAP expression upon nisin and theophylline induction in *L. lactis*. The resulting NICE-T7 system achieved a 2.8-fold increase in GFP expression compared to the original NICE system. Additionally, adenosine deaminase was fused to T7RNAP, generating the MutaT7LL system, which successfully mediated targeted gene mutagenesis in *L. lactis*. This work provides a versatile platform that enhances recombinant protein production and enables continuous targeted gene mutagenesis in *L. lactis*.

## 2 Results

### 2.1 Toxicity of T7RNAP in *E. coli* due to leaky expression from the PnisA promoter

**Fig. 1:**
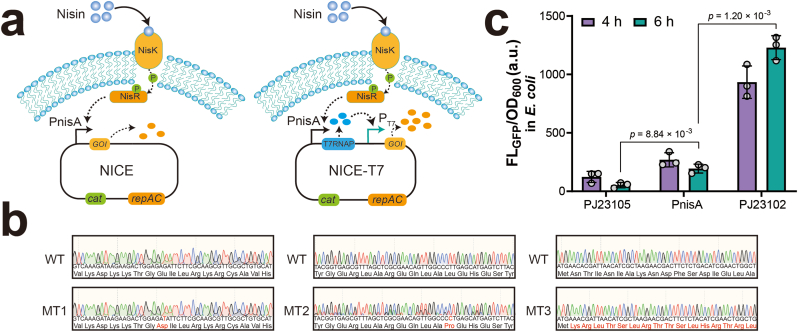
**T7RNAP leaky expression in *E. coli* from the *L.* lactis PnisA promoter. (a)** Schematic representation of the original NICE system and the modified system incorporating T7RNAP cascading. In the updated system, nisin activates the two-component system NisRK, which subsequently induces T7RNAP expression to drive overexpression of the gene of interest (GOI) under the T7 promoter. **(b)** Sequencing results of the original T7RNAP and the PnisA-controlled T7RNAP. Mutant nucleotides and amino acids are highlighted in red. WT, wild-type T7RNAP; MT, T7RNAP expressed under the T7 promoter. **(c)** Fluorescence intensities of strains expressing GFP under the control of the PJ23105, PnisA, and PJ23102 promoters. Data are presented as the mean with standard deviation from three independent biological replicates, and statistical analysis was performed using a two-tailed student *t*-test (apply to all subsequent experiments).

To enhance protein production in the NICE system, we introduced a T7RNAP cascade designed to amplify gene expression. In this system, nisin activates the NisKR two-component regulatory system, initiating T7RNAP transcription and thereby increasing the expression of downstream target genes (Fig. 1a). Based on the strategy, a high-copy plasmid carrying the pSH71 replicon derived from pNZ8148 was constructed in *E. coli* MC1061, as the plasmid does not replicate in *E. coli* DH5α or Trans1-T1 (Fig. S1). The construct carried the T7RNAP gene, amplified from T7 phage and placed under the control of the *L. lactis* PnisA promoter, along with a non-codon-optimized *gfp* reporter gene driven by the T7 promoter. However, sanger sequencing of three randomly selected clones revealed DNA mutations in all samples, including frameshift mutations and nonsynonymous substitutions of the T7RNAP gene (Fig. 1b). GFP expression driven by a strong promoter did not impact cell growth (Fig. S2a), indicating that the PnisA promoter was unintentionally active in *E. coli* MC1061, leading to basal T7RNAP expression that induced toxicity and genetic instability [28].

To verify this hypothesis, we assessed the activity of PnisA in *E. coli* by comparing it to two well-characterized *E. coli* constitutive promoters, J23105 (low strength) and J23102 (high strength) [29]. Each promoter was used to drive *gfp* expression, and relative fluorescence intensities were measured after 4 and 6 h of incubation. The results demonstrated that PnisA was indeed recognized by *E. coli* transcription machinery and not inducible in *E. coli*, displaying activity between that of the J23105 and J23102 promoters (Fig. 1c and S2b). These findings demonstrated that the PnisA promoter is partially active in *E. coli*, warranting modifications to reduce its leaky expression when used in constructs involving T7RNAP.

### 2.2 Reduction of PnisA leaky expression by incorporating a theophylline-dependent riboswitch

**Fig. 2:**
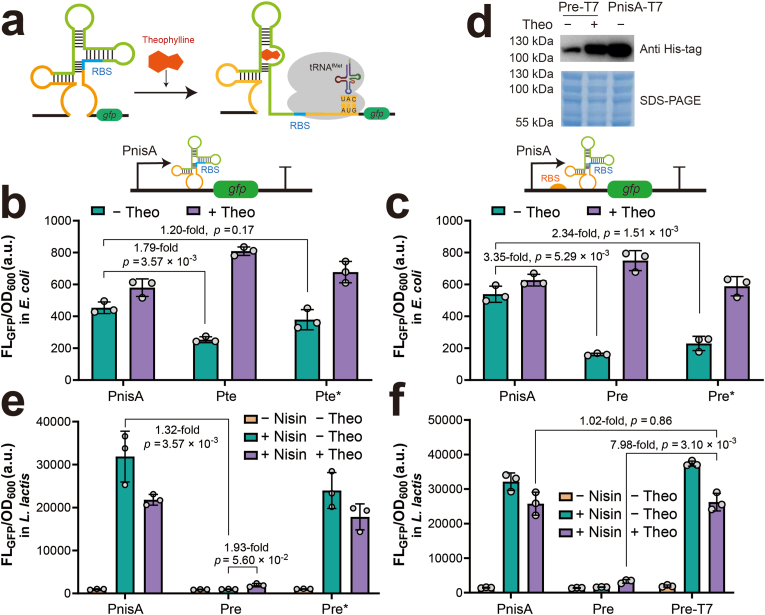
**Incorporation of a theophylline-dependent riboswitch reduces leaky expression from PnisA. (a)** Schematic illustration of the theophylline-dependent riboswitch mechanism. Theophylline induces a structural change in the aptamer, exposing the RBS for translation initiation. Fluorescence intensities of *E. coli* strains expressing GFP under the control of the PnisA, Pte, and Pte∗ promoters **(b)**, and the PnisA, Pre and Pre∗ promoters **(c)***.* Theo, theophylline. **(d)** Expression of the T7RNAP L39P variant driven by the Pre and PnisA promoters in *E. coli*. 5 mM theophylline was used to induce T7RNAP expression when indicated. **(e)** Fluorescence intensities of *L. lactis* strains expressing GFP under the PnisA, Pre, and Pre∗ promoters, measured under three different conditions. **(f)** Effect of T7RNAP cascading on GFP expression in *L. lactis*. Pre, GFP expression under the Pre promoter; Pre-T7, T7RNAP expression under the Pre promoter, with GFP expressed from the T7 promoter.

To address the leaky expression of PnisA, we integrated a theophylline-dependent riboswitch, a synthetic regulatory RNA that modulates translation initiation through conformational changes upon theophylline binding (Fig. 2a) [30,31]. Theophylline has high specificity to the theophylline-dependent riboswitches and is generally considered as safe at moderate concentrations [32]. We first evaluated the impact of theophylline on the growth and GFP expression of *E. coli* MC1061 and *L. lactis* NZ9000. Increasing theophylline concentrations from 1 mM to 20 mM substantially inhibited *E. coli* MC1061 growth while enhancing GFP fluorescence densities (Fig. S3a and b). In contrast, *L. lactis* NZ9000 growth remained unaffected at concentrations below 10 mM, although fluorescence densities decreased progressively with higher theophylline levels (Fig. S3c and d). Based on these observations, 5 mM theophylline was selected for subsequent experiments as it maintained cell viability while minimizing fluorescence interference.

To suppress PnisA leaky expression in *E. coli*, we selected two previously characterized theophylline-dependent riboswitches, RbxE and RbxE∗ [[33], [34], [35]], known to function across diverse gram-negative and gram-positive bacteria. Each riboswitch was integrated downstream of PnisA in two distinct configurations: (i) substituting the ribosome binding site (RBS) to create hybrid promoters Pte and Pte∗, and (ii) inserting between the RBS and the *gfp* start codon to generate Pre and Pre∗ (Fig. 2b and c). Fluorescence assays in *E. coli* demonstrated that all hybrid promoters reduced leaky expression in the absence of theophylline, with reductions ranging from 16 % to 70 % compared to the original PnisA promoter (Fig. 2b and c). Notably, the promoter Pre reduced the basal fluorescence intensity to 30 % of PnisA-driven expression without theophylline. Upon theophylline addition, riboswitch-mediated repression was relieved, and fluorescence levels returned to or exceeded those observed with the native PnisA promoter. To access T7RNAP expression under the control of the Pre and PnisA promoters, we fused a His-tag to the N-terminus of the T7RNAP L39P variant, an unintended mutant identified during plasmid construction, for detection by Western blot. The result confirmed that T7RNAP L39P was expressed under PnisA and that incorporation of the theophylline-dependent riboswitch largely reduced its basal expression (Fig. 2d). These findings demonstrated that both riboswitch integration strategies effectively minimized leaky expression while maintaining inducibility.

Considering the potential toxicity of T7RNAP overexpression in *L. lactis*, the two most effective hybrid promoters Pre and Pre∗ were further tested in *L. lactis* to evaluate riboswitch functionality. Interestingly, the promoter Pre∗ exhibited GFP expressed upon nisin induction even without theophylline, indicating ineffective repression. In contrast, the promoter Pre effectively suppressed GFP expression under the same condition, confirming that RbxE remained functional in *L. lactis* (Fig. 2e). However, the addition of theophylline only partially relieved RbxE-mediated repression, resulting in a 1.9-fold increase in fluorescence intensity (Fig. 2e). Finally, we attempted to construct the T7RNAP cascade plasmid using the optimized riboswitch configurations. Notably, only the construct in which PnisA was replaced by Pre was successfully assembled without mutations in *E. coli*, demonstrating that RbxE effectively suppressed leaky T7RNAP expression. After transformation into *L. lactis*, the engineered strain exhibited an 8.0-fold increase in fluorescence intensity compared to the strain without T7RNAP cascading, while showing a comparable fluorescence level to the original NICE system upon nisin and theophylline induction (Fig. 2f). These results indicate that further optimization of RbxE is required to enhance T7RNAP expression to achieve higher protein production.

### 2.3 High-throughput screening of optimized promoter Pre mutants

**Fig. 3:**
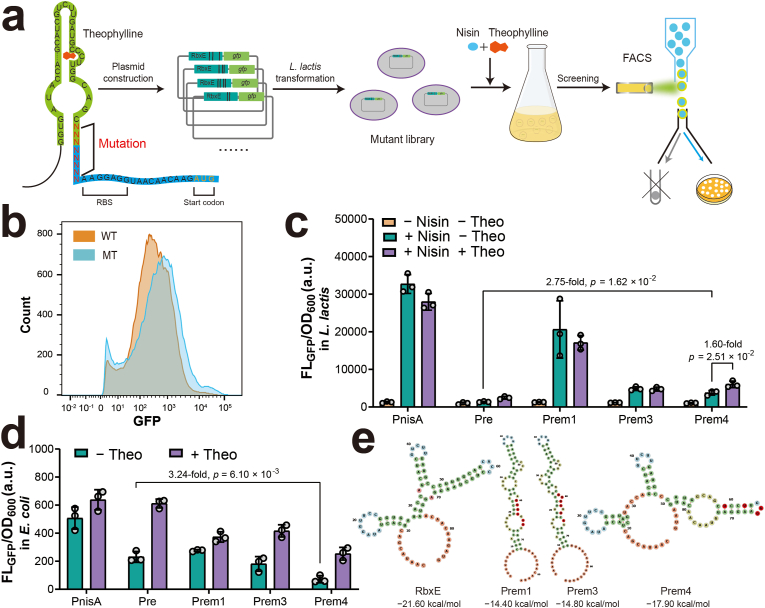
**Construction and screening of RbxE mutants with optimized promoter strength. (a)** Schematic diagram illustrating the workflow for constructing the RbxE mutant library and flow cytometry screening. **(b)** Fluorescence distribution of strains expressing GFP under the original Pre promoter and its mutant variants carrying RbxE mutations. WT, strain expressing GFP under the Pre promoter; MT, strain library containing RbxE mutations. **(c)** Fluorescence intensities of *L. lactis* strains RbxE mutants under three different conditions. Theo, theophylline. **(d)** Fluorescence intensities of *L. lactis* strains expressing GFP with T7RNAP cascading. **(e)** Predicted structures and thermodynamic properties of selected RbxE mutants.

Previous study has demonstrated that mutating the sequence between the riboswitch stem-loop and the ribosome binding site (RBS) can enhance protein expression following theophylline binding [36]. Building on this concept, we targeted the eight nucleotides immediately upstream of the RBS within the RbxE riboswitch region for mutagenesis. A degenerate primer containing eight random nucleotides (N) was used to generate a mutant library of the Pre promoter by PCR. The mutant library was introduced into *L. lactis* via electroporation, yielding approximately 8.12 × 10^4^ transformants. High-throughput screening was performed by sequentially inducing the library with nisin and theophylline, followed by fluorescence-activated cell sorting (FACS) to isolate the top 0.01 % of cells exhibiting fluorescence levels higher than those driven by the wild-type Pre promoter (Fig. 3a). FACS analysis confirmed that the mutant library displayed enhanced GFP fluorescence, particularly within the high-fluorescence subpopulation, compared to the original Pre promoter (Fig. 3b). A total of 100 individual cells from the top 0.01 % were selected for further verification (Fig. S4).

Ten colonies were randomly chosen, cultured, and assessed for promoter activity. All tested mutants exhibited higher promoter activity than the wild-type Pre upon nisin induction, even in the absence of theophylline (Fig. S5). However, only three mutants demonstrated further fluorescence enhancement following theophylline addition. These three, designated Prem1, Prem3, and Prem4, were subjected to shake flask cultivation for detailed characterization. Upon nisin induction, fluorescence intensities increased by 2.7- to 15-fold across these mutants. Notably, only Prem4 responded to theophylline addition, showing a further 1.6-fold increase in fluorescence (Fig. 3c). This indicated that mutations in Prem1 and Prem3 likely disrupted riboswitch functionality in *L. lactis*, whereas Prem4 retained the ability to regulate expression in response to theophylline. Further evaluation in *E. coli* demonstrated that GFP expression from all three mutants remained repressible by the riboswitches, with theophylline addition relieving the repression. Prem4, in particular, exhibited the lowest basal expression, reducing leaky expression to 30 % of the original Pre promoter level (Fig. 3d). Sequencing and RNA secondary structure prediction of the three mutants revealed that Prem4 carried 4 nucleotides changes and exhibited an enhanced predicted free energy relative to the wild-type RbxE riboswitch (Fig. 3e). This increase in free energy likely promotes the formation of a specific inhibitory structure that more effectively sequesters the ribosome binding site, thereby reducing background gene expression.

### 2.4 Construction and performance evaluation of the optimized NICE-T7 expression system

**Fig. 4:**
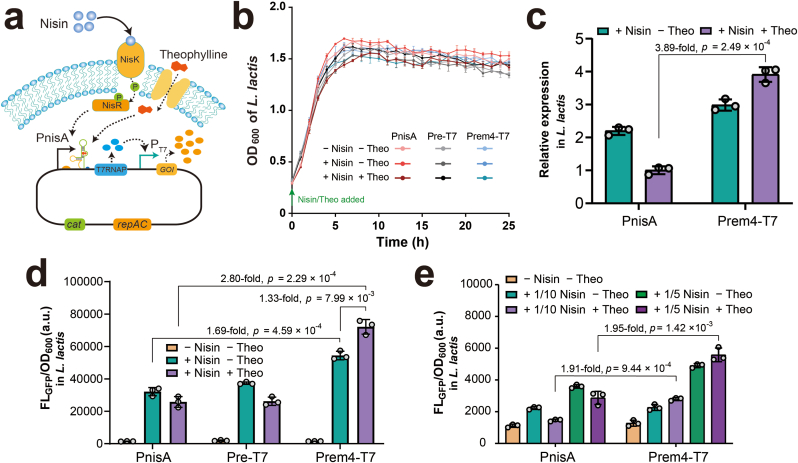
**Performance evaluation of the NICE-T7 system. (a)** Schematic representation of the NICE-T7 system. **(b)** Effect of T7RNAP expression on the growth of *L. lactis*. Pre-T7, strain with T7RNAP driven by the Pre promoter; Prem4-T7, strain with T7RNAP driven by the Prem4 promoter. Theo, theophylline. **(c)** Relative *gfp* transcription levels driven by the NICE and NICE-T7 systems. **(d)** Comparison of GFP expression between the NICE system and the upgraded systems incorporating T7RNAP cascading. **(e)** Effect of reduced nisin concentrations on GFP expression in the NICE and NICE-T7 systems. 1/5 Nisin and 1/10 Nisin correspond to 0.23 nM and 0.11 nM nisin, respectively.

Given the lower basal expression in *E. coli* and higher inducibility in *L. lactis*, the mutant promoter Prem4 was selected to optimize the NICE system. The resulting system, designated NICE-T7, incorporated T7RNAP expression driven by Prem4, activated upon nisin and theophylline induction (Fig. 4a). The plasmid was successfully constructed without introducing mutations in the T7RNAP gene, indicating that Prem4 effectively suppressed leaky expression and minimized T7RNAP-associated toxicity during cloning. The NICE-T7 plasmid was subsequently transformed into *L. lactis* NZ9000. To assess the impact of T7RNAP expression on host cell fitness, we compared the growth curves of strains carrying the original NICE system, the Pre-driven T7RNAP construct, and the Prem4-driven T7RNAP construct. Both T7RNAP-expressing strains exhibited similar cell densities with the strain lacking T7RNAP after nisin and theophylline induction (Fig. 4b), indicating that T7RNAP expression driven by Pre and Prem4 is not toxic to *L. lactis*.

qRT-PCR was performed to compare the *gfp* transcript levels between the NICE and NICE-T7 systems, revealing a 3.89-fold increase in *gfp* transcription in the NICE-T7 system (Fig. 4c). Subsequently, the protein expression capacity of the system was evaluated by measuring GFP fluorescence. The results showed comparable fluorescence levels between the original NICE system and the Pre-driven T7RNAP cascade, indicating insufficient T7RNAP expression to significantly enhance GFP production (Fig. 4d). In contrast, the Prem4-driven system produced 1.7-fold higher GFP fluorescence than the NICE system upon 11.3 nM nisin induction. The addition of 5 mM theophylline further increased GFP expression by 1.3-fold relative to nisin induction alone. Combined induction with nisin and theophylline resulted in a 2.8-fold increase in GFP fluorescence in the NICE-T7 system compared to the original NICE system (Fig. 4d), demonstrating enhanced protein expression efficiency. Notably, reducing the nisin concentration to 0.11 nM and 0.23 nM led to a significant decrease in GFP expression, highlighting the critical role of T7RNAP in enhancing protein production in the NICE-T7 system (Fig. 4e).

### 2.5 Construction of a targeted mutagenesis system in *L. lactis* based on the NICE-T7 platform

**Fig. 5:**
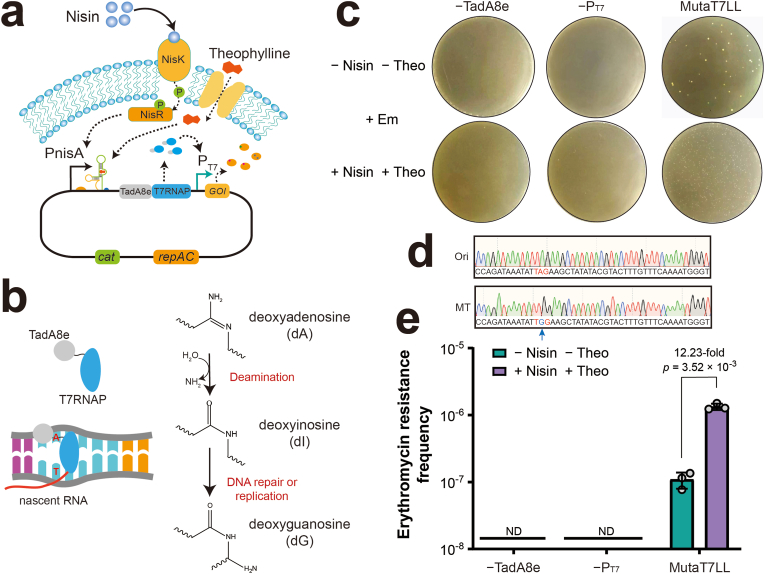
**Design and validation of the MutaT7LL system in *L. lactis*. (a)** Schematic representation of the MutaT7LL system in *L. lactis*. **(b)** Diagram illustrating the process of MutaT7LL-mediated A-to-G mutation. **(c)** Representative images showing the counts of erythromycin- and chloramphenicol-resistant colonies generated by the MutaT7LL system, as well as by systems lacking either Tada8e or the T7 promoter. **(d)** Sequencing results of the original *ermB*560A and *ermB*560G > A mutant conferring erythromycin resistance. **(e)** Comparison of erythromycin resistance frequency between the MutaT7LL system and the systems lacking either Tada8e or the T7 promoter. ND, not detected.

T7RNAP has been widely applied for targeted gene diversification by tethering base deaminases. TadA is a tRNA adenosine deaminase that catalyzes deamination of adenosine for inosine formation at position 34 in tRNA [37]. Through phage-assisted evolution, the enzyme was evolved to generate TadA8e, which operates on DNA with a relatively high editing efficiency [38,39]. Building on this foundation, we developed a targeted mutagenesis system in *L. lactis*, designated MutaT7LL, by fusing TadA8e to the N-terminus of T7RNAP within the NICE-T7 platform (Fig. 5a). Upon induction with nisin and theophylline, the expression of MutaT7LL was designed to mediate A-to-I transitions in the DNA coding strand downstream of the T7 promoter during transcription, with inosine subsequently recognized as guanosine during DNA replication or repair (Fig. 5b).

To evaluate the mutagenic efficiency of MutaT7LL, we constructed a reporter system based on the *ermB* gene, which encodes erythromycin resistance. Specifically, the “TGG” codon encoding Trp187 in *ermB* was mutated to the “TAG” stop codon, abolishing the erythromycin resistance. The *ermB* mutant was placed downstream of the T7 promoter, such that erythromycin resistance could only be restored if MutaT7LL reverted the “TAG” stop codon back to “TGG” through targeted A-to-G editing. The plasmid expressing both MutaT7LL and the *ermB* mutant was introduced into *L. lactis* NZ9000. Following induction with nisin and theophylline for 48 h, MutaT7LL expression had no detectable impact on host viability (Fig. S6a and b). Subsequently, we evaluated the efficiency of erythromycin resistance acquisition. A substantial number of resistant colonies were observed only in the presence of both MutaT7LL and the T7 promoter (Fig. 5c). In contrast, strains lacking MutaT7LL expression or the T7 promoter yielded no colonies on erythromycin-containing plates, confirming that both components were essential for targeted mutagenesis.

To verify the genetic basis of resistance restoration, individual erythromycin-resistant colonies were randomly selected, and the *ermB* gene was sequenced. The sequencing results confirmed successful A-to-G mutations that converted the “TAG” stop codon back to “TGG”, thereby restoring the functional *ermB* gene and erythromycin resistance (Fig. 5d). We further quantified the mutagenesis efficiency by calculating the erythromycin resistance frequency, which reached 1.33 × 10^−6^. This frequency was significantly higher than that observed in control strains lacking MutaT7LL or the T7 promoter (Fig. 5e). In addition, plasmids sequencing revealed two on-target mutations using the MutaT7 system, one of which was located at the stop codon, compared to a single mutation near the translation start codon in the negative control lacking TadA8e. These findings suggest that the MutaT7LL system has the potential for targeted gene mutagenesis in *L. lactis* (Fig. S6c and d).

## 3 Discussion

In the study, we integrated T7RNAP into the widely used NICE system in *L. lactis* to enhance protein production. T7RNAP and other transcriptional regulators, including HrpRS, ECF11_987, and LNV1 [[40], [41], [42]], have been employed as genetic amplifiers to improve biosensor sensitivity and expand dynamic range. Unlike other amplifiers, T7RNAP possesses inherently strong transcriptional activity, making it not only a potent signal amplifier but also a robust transcriptional machinery for protein overexpression [20]. These characteristics make T7RNAP a promising candidate for coupling with the NICE system to improve protein yields. However, a major challenge with T7RNAP is the need for tight expression control. Its excessive expression can lead to host toxicity by producing large amounts of mRNA, overloading the ribosome pool, generating misfolded proteins, and depleting cellular resources. Our results demonstrated that the *L. lactis* PnisA promoter is partially recognized by *E. coli* RNAP, leading to unintended basal expression of T7RNAP (Fig. 1c). This leakiness may be attributed to the similarity between the −10 (TACAAT) and −35 (ATTAAA) regions of PnisA and the conserved −10 (TATAAT) and −35 (TTGACA) motifs recognized by *E. coli* σ^70^ [43]. Consequently, leaky expression of T7RNAP, rather than GFP, in *E. coli* resulted in toxicity and loss-of-function mutations [28].

Several strategies have been developed to minimize T7RNAP leaky expression, including chromosomal integration to reduce gene copy number, regulation under inducible promoters like lacUV5 [44], co-expression of T7 lysozyme to inhibit basal activity [45], and medium optimization [46]. Riboswitches provide an additional layer of control by adopting alternative RNA conformations that either sequester the ribosome binding site (RBS) or terminate transcription in the absence of the ligand [47]. These RNA-based regulators enable rapid, efficient, and ligand-dependent gene control without the need for additional protein factors. The theophylline-dependent riboswitch offers several advantages for industrial applications: it exhibits minimal crosstalk with host metabolism, is functional in both gram-negative and gram-positive bacteria, and is cost-effective and well-tolerated by common bacteria [29,30].

Specifically, the theophylline-dependent riboswitch RbxE has been reported to function across diverse bacterial species, including *E. coli*, *B. subtilis* and *Parageobacillus thermoglucosidasius* [33,34]. In our study, RbxE effectively reduced PnisA leaky expression in *E. coli*. However, the hybrid promoter failed to achieve efficient activation in *L. lactis*, likely due to differences in RNA folding environments or RBS compatibility. Directed mutagenesis within the riboswitch between the adapter and RBS followed by high-throughput screening significantly improved riboswitch performance [36]. Incorporating a low copy-number replicon into the NICE-T7 plasmid may also decrease the leaky expression of T7RNAP, but its compatibility with the broad-host-range pSH71 replicon should be carefully considered. An alternative strategy to avoid T7RNAP leaky expression in *E. coli* is to construct the shuttle plasmid directly in *L. lactis*. However, the low transformation efficiency of *L. lactis* by electroporation significantly limits the size and diversity of DNA libraries, making large-scale library construction challenging. Incorporating the theophylline-dependent riboswitch effectively reduces leaky expression, enabling library construction in *E. coli*, which is more efficient and labor-saving.

The upgraded NICE-T7 system offers advantages over the traditional NICE system by incorporating T7RNAP regulated by a theophylline-dependent riboswitch. One of the key improvements is the enhanced protein production, with GFP expression increasing 2.8-fold compared to the original system (Fig. 4b). This enhancement is attributed to the strong and highly processive transcriptional activity of T7RNAP [10], which generates higher mRNA levels and, consequently, greater protein yields than the native *L. lactis* RNAP. Additionally, T7RNAP exhibits strict specificity for its T7 promoter [48,49], which effectively minimizes unintended interactions with *L. lactis* native promoters and reduces interference with host metabolic pathways. Importantly, cell growth harboring the NICE-T7 system following induction with nisin and theophylline remained comparable to the wild-type strain, indicating that the expression level of T7RNAP was not toxic to *L. lactis*. This suggests that the system could tolerate further optimization to enhance T7RNAP expression and potentially achieve higher protein production. Given that protein expression from the Prem4 promoter is lower than that from the original PnisA promoter, applying error-prone PCR to introduce mutations across the entire riboswitch, along with both positive and negative screening, may help identify variants with improved regulatory performance. Additionally, fine-tuning the RBS strength downstream of the riboswitch may further optimize translation initiation without compromising inducibility.

Leveraging the orthogonality between T7RNAP and its cognate promoter, we further developed the NICE-T7 system into the MutaT7LL system by fusing adenosine deaminase to T7RNAP, enabling targeted gene mutagenesis. The MutaT7LL system achieved an erythromycin resistance frequency of about 1.33 × 10^−6^, comparable to the MutaT7 system in *E. coli* [21]. This result demonstrates the system's effectiveness for continuous in vivo mutagenesis of target genes in *L. lactis*. Currently, MutaT7LL employs adenosine deaminase, limiting mutations primarily to adenosine residues, which may restrict its broader application. Previous studies have shown that fusing cytidine deaminase to T7RNAP enables efficient cytidine-targeted mutagenesis [23,50,51]. Future optimization of the MutaT7LL system could involve expressing additional T7RNAP chimeras fused to cytidine deaminase, as reported in *E. coli* [[52], [53], [54]]. Such modifications would expand the mutation spectrum and potentially increase the overall mutation frequency. To the best of our knowledge, MutaT7LL represents the first system developed for continuous in vivo gene mutagenesis in *L. lactis*. This platform offers a powerful tool for protein engineering and strain improvement, providing a more efficient alternative to traditional adaptive laboratory evolution approaches.

## 4 Conclusion

In this study, we developed the NICE-T7 system by integrating T7RNAP into the NICE system in *L. lactis*. T7RNAP expression was tightly regulated by a theophylline-dependent riboswitch in *E. coli* and induced by nisin and theophylline in *L. lactis*. The upgraded system enhanced GFP protein expression by 2.8-fold compared to the traditional NICE system. The MutaT7LL system was further developed by fusing adenosine deaminase to T7RNAP in the NICE-T7 system, enabling efficient A-to-G mutation and facilitating continuous in vivo mutagenesis of target genes in *L. lactis*.

## 5 Materials and methods

### 5.1 Bacterial strains, growth conditions, and plasmid construction

All bacterial strains and plasmids used in this study are listed in Tables S1 and S2. *E. coli* MC1061 was used as the host strain for plasmid construction and was cultured in Luria-Bertani (LB) medium (10 g/L tryptone, 5 g/L yeast extract, and 10 g/L NaCl) at 37 °C. *L. lactis* NZ9000 was grown at 30 °C in GM17 medium (M17 medium supplemented with 5 g/L glucose). The M17 medium (5 g/L soybean peptone, 2.5 g/L casein peptone, 5 g/L beef extract, 19 g/L sodium glycerophosphate, 0.25 g/L magnesium sulfate, 0.5 g/L sodium ascorbate, 2.5 g/L casein hydrolysate, 2.5 g/L yeast extract, and 5 g/L lactose, pH 7.2) was purchased from Solarbio Science & Technology Co., Ltd (Beijing, China). When required, cultures were supplemented with 25 μg/mL chloramphenicol, 11.3 nM nisin, or 5 mM theophylline. Unless otherwise specified, all concentrations remained constant throughout the study. The primers used in this study are listed in Table S3. Plasmid construction was performed using the pEASY-Basic Seamless Cloning and Assembly Kit from TransGen Biotech Co., Ltd. (Beijing, China).

### 5.2 Construction of plasmids encoding the NICE-T7 and MutaT7LL systems

To construct the pLZPE12 plasmid harboring the NICE-T7 system, a 2.9-kb DNA fragment containing the PnisA promoter, replication protein RepAC, and chloramphenicol resistance gene was amplified from the pNZ8148 plasmid to serve as the vector backbone. The T7RNAP gene was amplified from the *E. coli* BL21(DE3) genome, and the hybrid Prem4 promoter and *slpA* terminator were fused to its 5′ and 3’ ends, respectively, via overlap PCR. The *gfp* gene was synthesized by GENEWIZ Bio Inc. (Suzhou, China), with the T7 promoter incorporated at its 5′ end through overlap PCR. The vector backbone was then assembled with the purified DNA fragments encoding T7RNAP and *gfp* gene using the pEASY-Basic Seamless Cloning and Assembly Kit. The resulting reaction product was transformed into *E. coli* MC1061, and positive clones were confirmed by PCR and sequencing. For the construction of pLZPE13 plasmid encoding the MutaT7LL system, the gene encoding TadA8e was amplified from the pUC57-GDE1 plasmid. The *ermB* gene was amplified from pHSB04X, and the *ermB*560G > A mutation was introduced via overlap PCR. The pLZPE12 backbone, excluding the *gfp* gene, was assembled with the TadA8e-encoding DNA fragment and the *ermB*560G > A mutation-containing fragment using seamless cloning. Transformants were verified by PCR and sequencing.

### 5.3 Measurement of fluorescence density

For *E. coli*, overnight cultures were diluted in fresh LB medium and incubated for 4 h unless otherwise specified. Cells were harvested by centrifugation at 5000 rpm for 1 min, washed twice, and resuspended in phosphate-buffered saline (PBS; 8 g/L NaCl, 0.2 g/L KCl, 1.44 g/L Na_2_HPO_4_ and 0.24 g/L KH_2_PO_4_, pH 7.4). OD_600_ and fluorescence were measured using a Grating-based Multifunctional Microplate Reader (Infinite M200 Pro, Tecan Group Company, Swiss) with excitation at 488 nm and emission at 520 nm. Fluorescence density was calculated by normalizing fluorescence to OD_600_. For L. *lactis*, overnight culture was diluted in fresh GM17 medium and incubated at 30 °C for 1 h before the addition of 5 mM theophylline (when indicated). When cultures reached an OD_600_ of 0.15–0.25, 11.3 nM nisin was added to induce the PnisA promoter, and cells were harvested after 3 h of induction. OD_600_ and fluorescence were measured using the same protocol as for *E. coli*.

### 5.4 Analysis of T7RNAP expression

*E. coli* strains harboring T7RNAP expression plasmids were grown overnight, then diluted into fresh LB medium and incubated at 37 °C for 3 h. Where indicated, 5 mM theophylline was added, followed by an additional 1 h incubation. Cells were harvested by centrifugation at 13000 rpm for 1 min at 4 °C, resuspended in PBS, and lysed by sonication. The lysates were clarified by centrifugation, mixed loading sample buffer, and boiled at 95 °C for 5 min. Proteins were separated on 4–20 % Bis-Tris gel (Genscript, Nanjing, China) and transferred to PVDF membranes using a wet transfer system at 100 V for 90 min. Membrane was blocked with TBST containing 5 % skim milk for 1 h at room temperature and incubated overnight at 4 °C with anti-His antibody (1:4000 dilution). After washing three times with TBST, signals were visualized using an ECL detection kit (ABclonal, Wuhan, China).

### 5.5 Construction and screening of the RbxE mutant library

The RbxE mutant library was generated by introducing mutations into the eight nucleotides immediately upstream of the RBS using PCR with a degenerate primer containing eight randomized bases (N). The PCR product was phosphorylation using T4 polynucleotide kinase (NEB) and self-ligated with T4 DNA ligase (NEB). The ligation products were purified via isopropanol precipitation and electroporated into *E. coli* MC1061. To estimate the library size, a portion of the transformation mixture was serially diluted and plated on LB agar supplemented with chloramphenicol. The total number of *E. coli* transformants was approximately 1.23 × 10^5^, which were pooled for plasmid extraction and further transformation into *L. lactis* NZ9000. The total number of *L. lactis* transformants was estimated to be 8.12 × 10^4^, and all transformants were collected for subsequent screening.

For flow cytometry screening, *L. lactis* transformants containing the RbxE mutant library was collected, resuspended in GM17 liquid medium, and incubated at 30 °C with shaking at 250 rpm for 5 h. The culture was subsequently transferred to fresh GM17 medium and induced with 5 mM theophylline after 1 h of incubation. After an additional 2 h, 11.3 nM nisin was added, and induction continued for 3 h before flow cytometry analysis. The cells were then harvested, resuspended in PBS, and diluted to an OD_600_ of 0.03. Flow cytometry was performed using a BD FACSAria Fusion SORP flow cell sorter with an excitation wavelength of 488 nm and an emission wavelength of 520 nm. Cells exhibiting the top 0.01% fluorescence were isolated, and 100 individual cells were collected and plated on GM17 agar.

Following flow cytometry screening, ten single colonies were randomly selected and cultured in GM17 medium at 30 °C. Mutants exhibiting increased fluorescence intensity upon induction with both nisin and theophylline, compared to nisin alone, were selected for further analysis. The mutants were subsequently cultured in shaking flasks containing GM17 medium to quantify fluorescence intensity following nisin and theophylline induction. The plasmid from the final selected strain was extracted, transformed into *E. coli* MC1061, and analyzed for GFP leaky expression.

### 5.6 qRT-PCR analysis of *gfp* gene transcription

Overnight cultures were diluted into fresh GM17 medium and incubated at 30 °C for 1 h. Where indicated, 5 mM theophylline was added. When cultures reached an OD_600_ of 0.15–0.25, 11.3 nM nisin was added to induce the PnisA promoter. After 3 h of induction, cells were harvested by centrifugation at 13000 rpm for 1 min at 4 °C and snap-frozen in liquid nitrogen. Cell pellets were disrupted using sterile glass grinding rods and resuspended in TE buffer containing lysozyme. After incubation at 25 °C for 10min, total RNA was extracted using the RNeasy Mini Kit (Qiagen, Germany) according to the manufacturer's instructions. cDNA was synthesized using the EasyScript One-Step gDNA Removal and cDNA Synthesis SuperMix (TransGen Biotech, Beijing, China). qRT-PCR was performed using ChamQ Universal SYBR qPCR Master Mix (Vazyme Biotech, Nanjing, China) on a LightCycler® 96 system (Roche, Switzerland). The 16S rRNA gene was used as the internal reference for normalization.

### 5.7 Mutagenesis analysis of the erythromycin resistance gene

An overnight culture of *L. lactis* harboring the MutaT7LL system was transferred to fresh GM17 medium and induced with nisin and theophylline. After 48 h, cells were harvested by centrifugation at 5000rpm for 10 min, washed, and resuspended in PBS. The cell suspension was then diluted and plated onto GM17 agar containing either 25 μg/mL chloramphenicol or a combination of 25 μg/mL chloramphenicol and 10 μg/mL erythromycin. After 2–3 days of incubation, the number of single colonies on each plate was counted. The erythromycin resistance frequency was calculated by dividing the number of colonies resistant to both erythromycin and chloramphenicol by the total number of chloramphenicol-resistant colonies. Plasmids from erythromycin-resistant colonies were extracted and sequenced to identify mutations.

## CRediT authorship contribution statement

**Ying Huang:** Writing – original draft, Visualization, Methodology, Investigation, Formal analysis. **Kang Ma:** Validation, Investigation. **Yan Li:** Validation, Investigation. **Qingyan Li:** Supervision, Project administration. **Fuping Lu:** Supervision, Project administration. **Xueli Zhang:** Writing – original draft, Supervision, Project administration, Funding acquisition, Conceptualization. **Zhe Sun:** Writing – review & editing, Writing – original draft, Visualization, Supervision, Project administration, Funding acquisition, Data curation, Conceptualization.

## Funding

This work was financially supported by the Strategic Priority Research Program of the 10.13039/501100002367Chinese Academy of Sciences (XDC0110200), the 10.13039/501100001809National Natural Science Foundation of China (32270031), the 10.13039/501100014219National Science Foundation for Distinguished Young Scholars (32225031), 10.13039/501100001809NSFC Basic Science Center Program (32488301), the Tianjin Synthetic Biotechnology Innovation Capacity Improvement Project (TSBICIP-CXRC-064), and the Hundred Talents Program of the 10.13039/501100002367Chinese Academy of Sciences.

## Declaration of competing interest

The authors declare that they have no known competing financial interests or personal relationships that could have appeared to influence the work reported in this paper.
