# Supplementary material for: Engineering T7 RNA polymerase-cascaded systems controlled by nisin and theophylline for protein overexpression and targeted gene mutagenesis in Lactococcus lactis
Source: Synth Syst Biotechnol. 2025 Jun 22;10(4):1150–9. doi: 10.1016/j.synbio.2025.06.008 (PMC12269272; doi:10.1016/j.synbio.2025.06.008)
Supplement: Multimedia component 1 [file mmc1.pdf]

Supplementary information

Engineering T7 RNA polymerase-cascaded systems controlled by nisin and theophylline for protein overexpression and targeted gene mutagenesis in *Lactococcus lactis*

Ying Huang[a,b] , Kang Ma[a,b] , Yan Li[a,b] , Qingyan Li[b,c], Fuping Lu[a], Xueli Zhang[b,c,*], Zhe Sun[b,c,*]

[a]College of Biotechnology, Tianjin University of Science and Technology, Tianjin 300457, China
[b]Key Laboratory of Engineering Biology for Low-carbon Manufacturing, Tianjin Institute of Industrial Biotechnology, Chinese Academy of Sciences, Tianjin 300308, China
[c]National Center of Technology Innovation for Synthetic Biology, Tianjin 300308, China

*Correspondence:
Xueli Zhang, Tel: 022-84861983, Email: zhang_xl@tib.cas.cn
Zhe Sun, Tel: 022-24828790, Email: sunzhe@tib.cas.cn

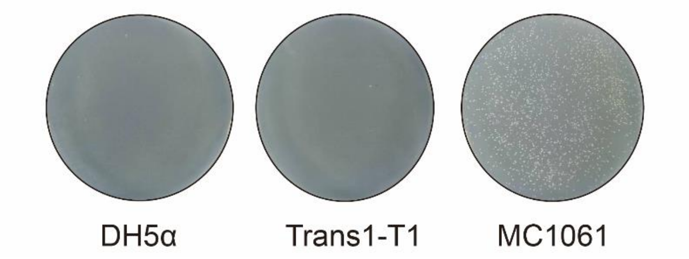

Figure S1. Replication capability of the plasmid carrying the pSH71 replicon in *E. coli* DH5α, Trans1-T1, and MC1061. Representative images showed colony formation following plasmid transformation.

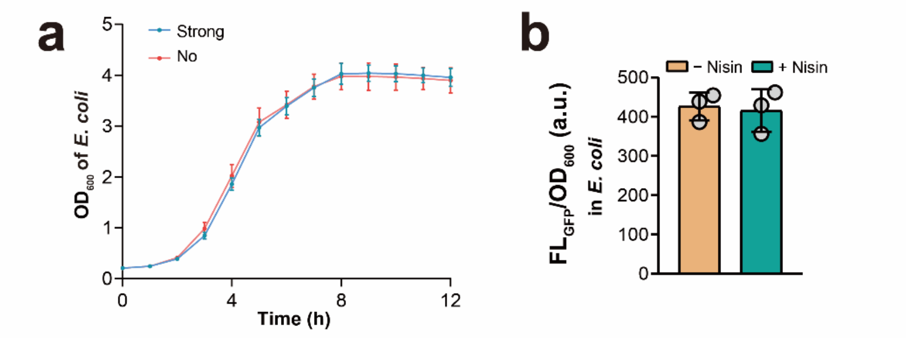

Figure S2. Evaluation of GFP toxicity and PnisA promoter inducibility in *E. coli* MC1061. (a) Growth curves of *E. coli* MC1061 strains harboring plasmids expressing GFP under either a strong promoter (J23119) or no promoter. **(b)** Column plot showing PnisA promoter activity in the presence and absence of nisin induction.

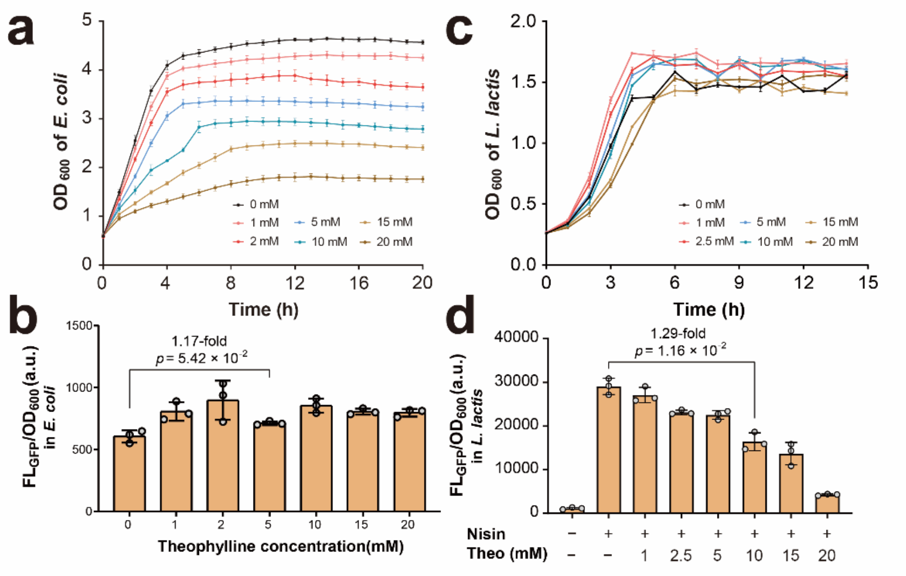

Figure S3. Effects of theophylline on the growth and fluorescence intensity of *E. coli* and *L. lactis*. Growth curves of *E. coli* MC1061 **(a)** and *L. lactis* NZ9000 **(c)** harboring the NICE system under varying theophylline concentrations (0–20 mM). Fluorescence intensities of *E. coli* MC1061 **(b)** and *L. lactis* NZ9000 **(d)** expressing GFP under the control of the PnisA promoter.

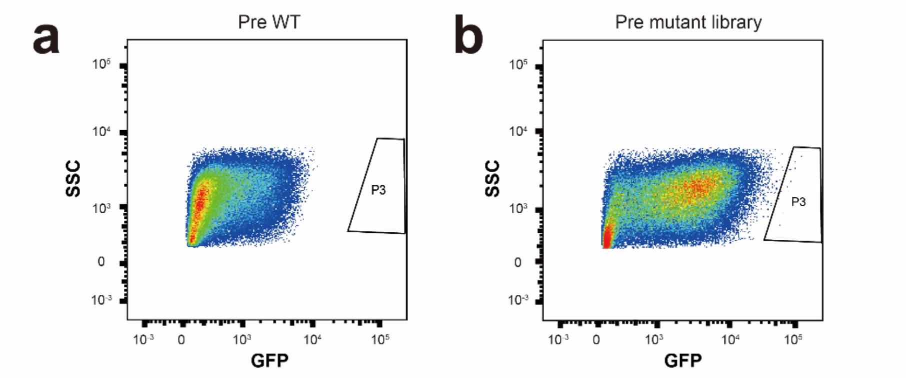

Figure S4. Flow cytometry-based screening of the Pre promoter mutant library. **(a)** Fluorescence intensity of *L. lactis* expressing GFP under the original Pre promoter, served as the negative control. **(b)** Fluorescence intensity of *L. lactis* expressing GFP from the Pre promoter mutant library. Gating criteria were defined based on the fluorescence profile of the negative control.

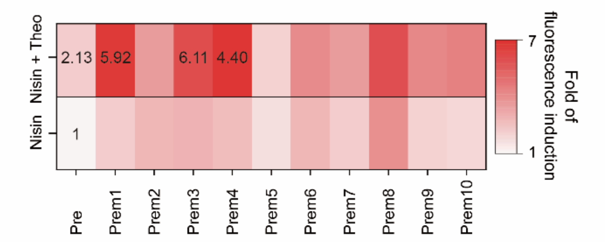

Figure S5. Fluorescence induction analysis of Pre promoter mutants following flow cytometry screening. The fluorescence intensity of the strain expressing GFP under the Pre promoter was normalized to 1 as a control. Each column represents a strain carrying a mutant Pre promoter, with individual cells displaying the fold change in fluorescence induction under specific conditions relative to the control.

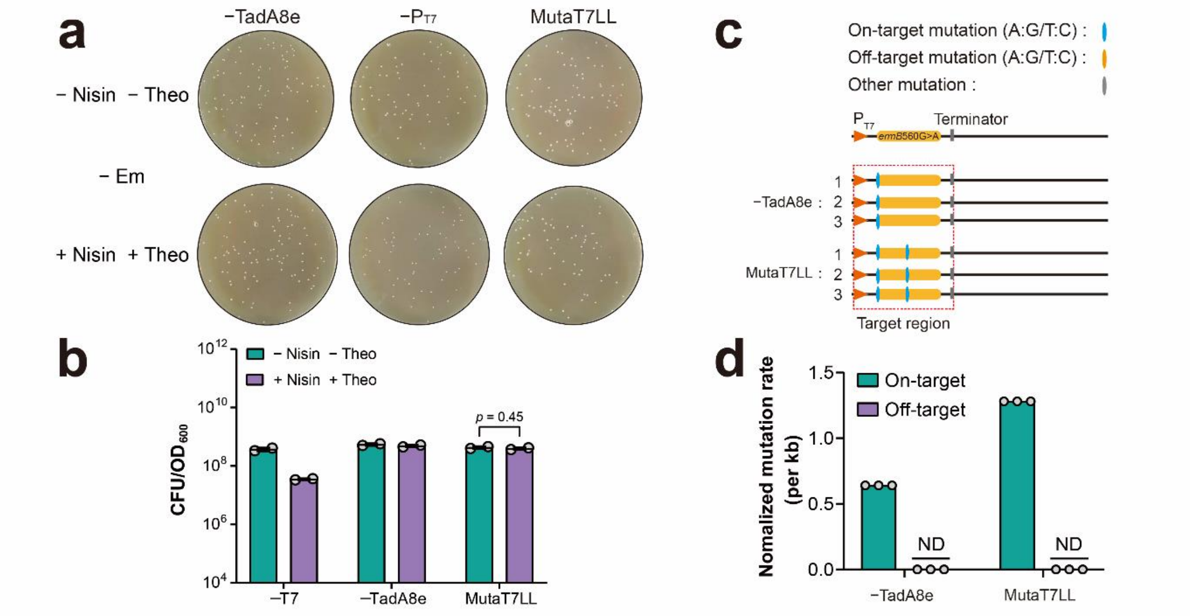

Figure S6. Viability and mutagenesis analysis of *L. lactis* strains harboring the MutaT7LL system. Representative images **(a)** and column plot **(b)** showing the counts of chloramphenicol-resistant colonies from strains expressing the full MutaT7LL system or variants lacking either TadA8e or the T7 promoter, under both uninduced and induced conditions. On-target and off-target mutations **(c)**, and normalized mutation rate **(d)** in *L. lactis* strains harboring the MutaT7LL system compared to the control strain lacking TadA8e.

Supplementary Table 1. Strains used in this study

Strain	Description	Reference
E. coli MC1061	Wild-type *Escherichia coli*	Lab stock
E. coli BL21(DE3)	For obtaining T7RNAPsequence	Lab stock
Pte	MC1061 harboring plasmid pLZPE06	This study
Pte*	MC1061 harboring plasmid pLZPE08	This study
Pre	MC1061 harboring plasmid pLZPE07	This study
Pre*	MC1061 harboring plasmid pLZPE09	This study
PnisA	NZ9000 harboring plasmid pLZPE01	This study
PJ23105	NZ9000 harboring plasmid pLZPE03	This study
PJ23102	NZ9000 harboring plasmid pLZPE04	This study
L.lactis NZ9000	NICE expression system host	Lab stock
Pre-T7	NZ9000 harboring plasmid pLZPE10	This study
Prem-4	NZ9000 harboring plasmid pLZPE11	This study
Prem4-T7	NZ9000 harboring plasmid pLZPE12	This study
MutaT7LL	NZ9000 harboring plasmid pLZPE13	This study
MutaT7(−TadA8e)	NZ9000 harboring plasmid pLZPE14	This study
MutaT7(−P_{T7})	NZ9000 harboring plasmid pLZPE15	This study
PnisA-his-T7	MC1061 harboring plasmid pLZPE16	This study
Pre-his-T7	MC1061 harboring plasmid pLZPE17	This study

Supplementary Table 2. Plasmids used in this study

Plasmids	Relevant characteristics	Reference
pNZ8148	nisA promoter, Cm^R, shuttle vector between *E. coli* and *L.lactis*	[1]
pHSB04X	Ery^R, shuttle vector between *E. coli* and *L.lactis*	[2]
pUC57_GDE1	Amp^R, GDE1 gene	Laboratory storage
pUC57_GFP	Amp^R, *gfp* gene	GENEWIZ
pLZPE01	pNZ8148 derivative harboring *gfp*, under the control of nisA promoter, for expression of *gfp*.	This study
pLZPE02	pLZPE01 derivative harboring T7RNAP, under the control of nisA promoter, and T7 promoter for expression of *gfp*.	This study
pLZPE03	pNZ8148 derivative harboring *gfp*, under the control of J23102 promoter, for expression of *gfp*.	This study
pLZPE04	pNZ8148 derivative harboring *gfp*, under the control of J23105 promoter, for expression of *gfp*.	This study
pLZPE05	pLZPE01 derivative harboring *gfp*, no promoter for expression of *gfp*.	This study
pLZPE06	pLZPE01 derivative harboring RbxE Riboswitch, substituting the ribosome binding site of PnisA to create hybrid promoter Pte, for expression of *gfp*.	This study
pLZPE07	pLZPE01 derivative harboring harboring RbxE Riboswitch, inserting between the RBS and the *gfp* start codon to generate hybrid promoter Pre, for expression of *gfp*.	This study
pLZPE08	pLZPE01 derivative harboring RbxE* Riboswitch, substituting the ribosome binding site of PnisA to create hybrid promoter Pte*, for expression of *gfp*.	This study
pLZPE09	pLZPE01 derivative harboring RbxE* Riboswitch, inserting between the RBS and the *gfp* start codon to generate hybrid promoter Pre*, for expression of *gfp*.	This study
pLZPE10	pLZPE10 derivative harboring T7RNAP, under the control of Pre promoter, for expression of for expression of T7RNAP, T7 promoter for expression of *gfp*.	This study
pLZPE11	pLZPE01 derivative harboring harboring RbxEM-4 Riboswitch mutant, inserting between the RBS and the *gfp* start codon to generate hybrid promoter Prem-4, for expression of *gfp*.	This study
pLZPE12	pLZPE10 derivative harboring T7RNAP, under the control of Prem-4 promoter, for expression of for expression of T7RNAP, T7 promoter for expression of *gfp*.	This study
pLZPE13	pLZPE12 derivative harboring fusion of TadA8e and T7RNAP, under the control of Prem-4 promoter for expression of TadA8e and T7RNAP, T7 promoter for expression of *ermB560G>A* mutant.	This study
pLZPE14	pLZPE12 derivative harboring T7RNAP, under the control of Prem-4 promoter, T7 promoter for expression of *ermB560G>A* mutant.	This study
pLZPE15	pLZPE11 derivative harboring fusion of TadA8e and T7RNAP, under the control of Prem-4 promoterfor expression of TadA8e and T7RNAP, no T7 promoter for expression of *ermB560G>A* mutant.	This study
pLZPE16	pLZPE02 derivative harboring fusion of his tag and T7RNAP, under the control of nisA promoter, and T7 promoter for expression of *gfp*.	This study
pLZPE17	pLZPE10 derivative harboring fusion of his tag and T7RNAP, under the control of Pre promoter, for expression of for expression of T7RNAP, T7 promoter for expression of *gfp*.	This study

Supplementary Table 3. Primers used in this study

Primer	Sequence
PnisA-*gfp*-F	GGAGGCACTCACCATGGGTAAGGGAGAAGAACTTTTCACTGGAG
PnisA-*gfp*-R	CTTACCCATGGTGAGTGCCTCCTTATAATTTATTTTGTAGTTCC
gfp-F	CACATGGCATGGATGAACTATACAAATAATTTCTTTGAACCAAAAT TAGAAAACCAAGGCTTG
gfp-R	GTATAGTTCATCCATGCCATGTGTAATCCC
PnisA-T7RNAP-F	CAAAATAAATTATAAGGAGGCACTCAATGAACACGATTAACATCGC TAAGAACGAC
PnisA-T7RNAP-R	CGTGTTCATTGAGTGCCTCCTTATAATTTATTTTGTAGTTCCTTCG
slpA terminator-spacer-*gfp*-F	GTAATACGACTCACTATAGGGAGAAAGGAGGCACTCACCATGGGT AAG
slpA terminator-spacer-*gfp*-R	GCCTCCTTTCTCCCTATAGTGAGTCGTATTACTCCCATTATATCTTTT TTTGCACTGATTGGTG
PJ23102-*gfp*-F	TTGACAGCTAGCTCAGTCCTAGGTACTGTGCTAGCAAGGAGGCACT CACCATGGGTAAGGGAG
PJ23102-*gfp*-R	CCCATGGTGAGTGCCTCCTTGCTAGCACAGTACCTAGGACTGAGCT AGCTGTCAAATCTGGAGCTG
cat-PJ23102-F	GTCACTAACCTGCCCCGTTAGTTGAAGAAGGTTTTTATATTACAGCT CCAGATTTGACAGCTAGCTCAGTCCTAGGTACTGTG
cat-J23102-R	CCTTCTTCAACTAACGGGGCAGGTTAGTGAC
PJ23105-*gfp*-F	GCTAGCAAGGAGGCACTCACCATGGGTAAGGGAGAAGAACTTTTC ACTGGAG
PJ23105-*gfp*-R	CCCATGGTGAGTGCCTCCTTGCTAGCATAGTACCTAGGACTGAGCT AG
cat-PJ23105-F	GATTTTACGGCTAGCTCAGTCCTAGGTACTATGCTAGCAAGGAGGC ACTCACCATGGGTAAGGGAG
cat-PJ23105-R	CCTTCTTCAACTAACGGGGCAGGTTAGTGAC
Pte-F	TGATACCAGCATCGTCTTGATGCCCTTGGCAGCACCCTGCTAAGGA GGTAACAACAAGATGGGTAAGGGAGAAGAACTTTTCACTGGA
Pte-R	CATCAAGACGATGCTGGTATCACCGGAACCTATAGTGAGTCGTATT GCGAACGAAATCATTGTATCTAACAAACTTCAGAATTTAATC
Pte*-F	CCCTTGGCAGCACCCTGCTAAGGAGGCAACAAGATGGGTAAGGGA GAAGAACTTTTCACTGGAG
Pte*-R	CATCAAGACGATGCTGGTATCACCGGAACCTATAGTGAGTCGTATT GCGAACGAAATCATTGTATCTAACAAACTTCAGAATTTAATC
Pre-F	TGATACCAGCATCGTCTTGATGCCCTTGGCAGCACCCTGCTAAGGA GGTAACAACAAGATGGGTAAGGGAGAAGAACTTTTCACTGGAG
Pre-R	CATCAAGACGATGCTGGTATCACCGGAACCTATAGTGAGTCGTATT GCGAACGAAATCATTGTATCTAACAAACTTCAGAATTTAATC
Pre*-F	GGTGATACCAGCATCGTCTTGATGCCCTTGGCAGCACCCTGCTAAG GAGGCAACAAGATGGGTAAGGGAGAAGAACTTTTCACTGGAG
Pre*-R	CATCAAGACGATGCTGGTATCACCGGTACCAATTGTGAGCGTATCC TCCTTATAATTTATTTTGTAGTTCCTTCGAACG
Pre-T7RNAP-F	GCACCCTGCTAAGGAGGTAACAACAAGATGAACACGATTAACATC GCTAAGAACGAC
Pre-T7RNAP-R	CGTGTTCATCTTGTTGTTACCTCCTTAGCAGGGTGC
T7RNAP-*eryB*-F	CACTATAGGGAGAAAGGAGGCACTCACCATGAACAAAAATATAAA ATATTCTCAAAACTTTTTAACGAGTGAAAAAG
T7RNAP-*eryB*-R	CTCACTATAGGGAGAAAGGAGGCACTCACC
*eryB*mutant-F	GTTCCAGATAAATATTAGAAGCTATATACGTACTTTGTTTCAAAATG GGTCAATC
*eryB*mutant-R	GAAACAAAGTACGTATATAGCTTCTAATATTTATCTGGAACATCTG

	TGGTATGGCGGGTAAG
Prem4-TadA8e-F	CCCTGCTAAGGAGGTAACAACAAGATGGGCAGCAGCTACCCATAC GACGTACCAGATTACGCTAGTGAAGTAGAGTTCAGCCATGAATA
Prem4-TadA8e-R	CTTGTTGTTACCTCCTTAGCAGGGTG
TadA8e-T7RNAP-F	CCCCAGGCACCAGCGAGAGCGCAACGCCGGAAAGCTCTGGTGGCA GCAGCGGCGGCTCCATGAACACGATTAACATCGCTAAGAACGAC
TadA8e-T7RNAP-R	GCGTTGCGCTCTCGCTGGTGCCTGGGGTTTCGGAACCCGAACTGCC ACCGCTAGAACCACCAGAGTTGATGGAGCTCTGGGCCTTCTTCT
eryB-terminator-F	CTTTGAACCAAAATTAGAAAACCAAGGCTTGAAACG
eryB-terminator-R	CAAGCCTTGGTTTTCTAATTTTGGTTCAAAGAAATTAAGAATAAAA CGGCTCTTTTATACGTAAAGGACG
T7RNAP-*slpA* terminator-spacer-F	CTTCGCGTTCGCGTAATGAAAAAGGCAGAGCGAAAGCTCTGTCTTT TTTCACGCCAATAAAAAACGGTCGCG
T7RNAP-*slpA* terminator-spacer-R	CGCTCTGCCTTTTTCATTACGCGAACGCGAAGTCCGACTCTAAG
rrnB T1 terminator-rrnB T2 terminator-*gfp*-F	GCATGCAGATTGCAGCATTACACGAGATATGGGTAAGGGAGAAGA ACTTTTCACTGGAGTTG
rrnB T1 terminator-rrnB T2 terminator-*gfp*-R	CGTGTAATGCTGCAATCTGCATGCAAG
cat-rrnB T1 terminator-rrnB T2 terminator-F	CCTGCCCCGTTAGTTGAAGAAGGTTTTTATATTACAGCTCCCAAATA AAATGAAAGGCTCAGTCGAAAGACTG
cat-rrnB T1 terminator-rrnB T2 terminator-R	CCTTCTTCAACTAACGGGGCAGG
PnisAYZ-F	CTAGTCTTATAACTATACTGACAATAGAAACATTAACAAATCTAAA ACAGTC
*gfp*YZ-R	CTTGAAGAAGTCGTGCCGCTTCATATG
Pte-YZ-F	GCAATACGACTCACTATAGGTTCCGGTG
Pre-YZ-F	CAAAATAAATTATAAGGAGGCAATACGACTCAC
RbxEM-4-T7RNAP-F	CATCGTCTTGATGCCCTTGGCAGCNNNNNNNNAAGGAGGTAAC
RbxEM-4-T7RNAP-R	CTGGTATCACCGGAACCTATAGTGAGTCG
terminator-P_{T7}-F	GTAATACGACTCACTATAGGGAGAAAGGAGGCACTCACCATGGGT AAG
terminator-P_{T7}-R	GCCTCCTTTCTCCCTATAGTGAGTCGTATTACTCCCATTATATCTTTT TTTGCACTGATTGGTG
T7RNAP-*eryB*-F	ATGAACAAAAATATAAAATATTCTCAAAACTTTTTAACGAGTGAAA AAG
T7RNAP-*eryB*-R	CTCCCATTATATCTTTTTTTGCACTGATTGGTG
PnisA-RbxEM-4-F	AGGAGGCAATACGACTCACTATAGGTTCCGGTGATACCAG
16SrRNA-F	GTGTCGTGAGATGTTGGGTT
16SrRNA-R	ATAAGGGGCATGATGATTTG
gfp-F1	TGTTCCTTGGCCAACACTTG
gfp-R1	GCACGTGTCTTGTAGTTCCC
PnisA-his-T7RNAP-F	CTCACCATGCACCACCATCATCATCATATGAACACGATTAACATCG CTAAGAACGAC
PnisA-his-T7RNAP-R	GTTCATATGATGATGATGGTGGTGCATGGTGAGTGCCTCCTTATAAT TTATTTTGTAGTTCCTTCG
Pre-his-T7RNAP-F	GATGCACCACCATCATCATCATATGAACACGATTAACATCGCTAAG AACGAC
Pre-his-T7RNAP-R	CGTGTTCATATGATGATGATGGTGGTGCATCTTGTTGTTACCTCCTT AGCAGGGTGC

Supplementary Table 4. Sequences of RbxE, RbxE*, and TadA8e used in this study

Gene	Sequence
RbxE	CAATACGACTCACTATAGGTTCCGGTGATACCAGCATCGTCTTGATGCCCTTGGCA<br>GCACCCTGCTAAGGAGGTAACAACAAGATG
RbxE*	ATACGCTCACAATTGGTACCGGTGATACCAGCATCGTCTTGATGCCCTTGGCAGCA<br>CCCTGCTAAGGAGGCAACAAGATG
TadA8e	ATGGGCAGCAGCTACCCATACGACGTACCAGATTACGCTAGTGAAGTAGAGTTCA<br>GCCATGAATATTGGATGCGTCATGCCTTGACTCTGGCGAAGCGCGCGCGTGACGAG<br>CGCGAGGTCCCTGTGGGAGCCGTGCTGGTGCTGAACAATAGAGTGATCGGCGAGG<br>GCTGGAACAGAGCGATCGGCCTGCACGACCCAACAGCCCATGCCGAAATTATGGC<br>CCTGAGACAGGGCGGCCTGGTCATGCAGAACTACAGACTGATTGACGCCACCCTGT<br>ACGTGACATTCGAGCCTTGCGTGATGTGCGCCGGCGCCATGATCCATTCTAGGATC<br>GGCCGCGTGGTGTTTGGCGTGAGGAACTCAAAAAGAGGCGCCGCAGGCTCCCTGA<br>TGAACGTGCTGAACTACCCCGGCATGAATCACCGCGTCGAAATTACCGAGGGAAT<br>CCTGGCAGATGAATGTGCCGCCCTGCTGTGCGATTTCTATCGGATGCCTAGACAGG<br>TGTTCAATGCTCAGAAGAAGGCCCAGAGCTCCATCAACTCTGGTGGTTCTAGCGGT<br>GGCAGTTCGGGTTCCGAAACCCCAGGCACCAGCGAGAGCGCAACGCCGGAAAGCT<br>CTGGTGGCAGCAGCGGCGGCTCC

References

1. Mierau I, Kleerebezem M. 10 years of the nisin-controlled gene expression system (NICE) in *Lactococcus lactis*. Appl Microbiol Biot 2005;68(6):705-17. https://doi.org/10.1007/s00253-005-0107-6.

2. Huang H, Song X, Yang S. Development of a RecE/T-assisted CRISPR-Cas9 toolbox for *Lactobacillus*. Biotechnol J 2019;14(7):e1800690. https://doi.org/10.1002/biot.201800690.
